# Fluoropyrimidine-Induced Severe Toxicities Associated with Rare DPYD Polymorphisms: Case Series from Saudi Arabia and a Review of the Literature

**DOI:** 10.3390/clinpract11030062

**Published:** 2021-07-27

**Authors:** Nedal Bukhari, Abdulraheem Alshangiti, Emad Tashkandi, Mohammed Algarni, Humaid O. Al-Shamsi, Hamoud Al-Khallaf

**Affiliations:** 1 Department of Medical Oncology, King Fahad Specialist Hospital, Dammam 31444, Saudi Arabia; Dralshangiti@gmail.com; 2 Department of Internal Medicine, Imam Abdulrahman Bin Faisal University, Dammam 34212, Saudi Arabia; 3 Oncology Centre, King Abdullah Medical City, Makkah 24246, Saudi Arabia; Emad_tashkandi@hotmail.com; 4 College of Medicine, Umm Al-Qura University, Makkah 24211, Saudi Arabia; 5 Oncology Department, King Abdulaziz Medical City, Riyadh 11426, Saudi Arabia; Garnimo2@gmail.com; 6 King Saud bin Abdulaziz University for Health Sciences, Riyadh 11481, Saudi Arabia; 7 Department of Oncology, Burjeel Cancer Institute, Burjeel Medical City, Abu Dhabi 999041, United Arab Emirates; humaid.al-shamsi@medportal.ca; 8 Emirates Oncology Society, Dubai 22107, United Arab Emirates; 9 College of Medicine, University of Sharjah, Sharjah 999041, United Arab Emirates; 10 Department of Pathology and Laboratory Medicine, King Fahad Specialist Hospital, Dammam 31444, Saudi Arabia; drhhk@hotmail.com

**Keywords:** dihydropyrimidine dehydrogenase, 5-Fluorouuracil, chemotherapy, DPYD, polymorphism

## Abstract

Dihydropyrimidine dehydrogenase (DPD) is the major enzyme in the catabolism of 5-Fluorouracil (5-FU) and its prodrug capecitabine. We report cases from our institute with colorectal cancer who experienced severe toxicities to standard dose 5-FU based chemotherapy. *DPYD* gene sequencing revealed rare different polymorphisms that prompted dose adjustments of administered 5-FU and capecitabine. To our knowledge, this is the first case series looking at *DPYD* polymorphisms in the Saudi Arabian population.

## 1. Introduction

5-FU has been the backbone of chemotherapy used in many different cancers since the late 1950s [1]. Capecitabine is an oral prodrug and has also been approved for medical use since 1998 [2]. Both drugs are widely prescribed and generally well-tolerated [3].

DPD is the initial and rate-limiting enzyme in the catabolism of 5-FU. More than 80% of the administered doses of 5-FU and capecitabine are rapidly degraded by DPD to inactive compounds within 24 h [3,4,5].

Complete or intermediate deficiency of DPD can result in severe, life-threatening side effects, including severe mucositis, diarrhea, and pancytopenia. Genetic polymorphism in its encoding gene *DPYD* has an established impact on enzyme level, and toxicity risks [3,4].

## 2. Case 1

A 65-year-old female referred to our academic institute with a diagnosis of locally advanced distal rectal adenocarcinoma staged at T2N2 underwent neoadjuvant long course concurrent chemoradiation with capecitabine at 625 mg/m^2^ twice daily on radiation days. She required an abdominoperineal resection (APR) and end colostomy. She was found to have a left lower lobe metastatic lesion, resected 6 weeks following her APR.

The patient initially received a XELOX regimen, with capecitabine at 1000 mg/m^2^ twice daily. Few days after starting treatment, she developed Grade III diarrhea, GIII fatigue, and GII mucositis reported as per Common Terminology Criteria for Adverse Events (CTCAE criteria). Capecitabine was stopped on day eight of treatment. Grade II pancytopenia was also noted on her laboratory investigations.

She was switched to a Q 2 weekly FOLFOX-6 regimen where she received a bolus of 5-FU, dosed at 400 mg/m^2^ on the first day of treatment, leucovorin, and oxaliplatin—followed by a 46 h of 5-FU infusion at 2400 mg/m^2^. Four days after completion of 5-FU infusion, she developed GIII mucositis, GIII diarrhea, and fever, for which she required hospital admission. She was clinically dehydrated, septic, with GIII pancytopenia on her CBC.

She also developed alopecia and phlebitis immediately after receiving one cycle of FOLFOX.

The patient required intravenous (IV) antibiotics and IV rehydration during her hospital stay. After obtaining informed consent, a blood sample was sent to Mayo Clinic for full *DPYD* gene sequencing. Result confirmed heterozygosity for c.2434G>A, rs371313778. Her report stated the following:

- Variation identified: Het rs371313778 Genomic position: Chr1: 97700416;
- cDNA change: c.2434G>A (Heterozygous) Amino acid change: p.Val812lle.

The patient’s symptoms and blood count improved in 10 days. The following cycle of chemotherapy was restarted after 2 weeks of rest. The dose of 5-FU chemotherapy was reduced by 50% with no change made to the oxaliplatin dose. 5-FU dose was gradually increased by 5% in subsequent cycles. Sixty percent of the standard dose of 5-FU was tolerated without severe toxicities.

## 3. Case 2

The second is a 64-year-old female, a cousin of case 1, who had a diagnosis of stage III colon adenocarcinoma, sigmoid primary, underwent a left hemicolectomy without complications.

She was found to have T4N2 disease, and the decision was made to start adjuvant treatment. Based on her family history of severe toxicity to 5-FU chemotherapy secondary to DPD deficiency, screening for DPYD polymorphism was done, and she was started on adjuvant XELOX at 40% dose-reduction in her capecitabine. The only side effect she reported was G1 diarrhea and mucositis, which was successfully managed with antidiarrheals and 2% oral viscous lidocaine. There were no unexpected changes in her CBC parameters.

A whole gene sequencing was performed at the CGC genetics lab. Additionally, the variant c.1601G>A p.(Ser534Asn), also known as DPYD*4, was detected in apparent homozygosity (Table 1).

A trial of increasing the capecitabine dose by 5–10% was done. Shortly after completion of her second cycle of capecitabine, she developed GIII diarrhea, mucositis, and pancytopenia and eventually required short hospital admission for IV rehydration. We decided to continue adjuvant treatment with no changes made to the dose she received on the first cycle of chemotherapy, which was 60% of the standard dose of capecitabine. No changes were made to the oxaliplatin dose.

## 4. Case 3

The third case regards a previously healthy 66-year-old male patient with a diagnosis of a T3N1 upper rectal moderately differentiated adenocarcinoma. He was planned for long-course concurrent chemoradiation (CCRT), and capecitabine at 625 mg/m^2^ twice daily was given on radiation (RT) days only. He developed diarrhea during the third week of treatment and was managed with loperamide. In the middle of the fourth week of CCRT, he experienced a decrease in his energy levels, his diarrhea worsened to 7 times/daily (GIII), and he was clinically dehydrated, which required hospital admission. A repeat blood work showed a grade IV neutropenia with WBC: 0.59 × 10^9^/L, neutrophil count: 0.27 × 10^9^/L, HGB: 12.1 g/dL, and a platelet count of 136 × 10^9^/L. Treatments were put on hold, and intravenous rehydration was given to him throughout his 10-day stay in hospital. His oncologist elected to put his chemotherapy on hold throughout his neoadjuvant radiation treatment course. The patient completed his neoadjuvant radiation without experiencing the side effects reported with radiation.

A whole gene sequencing was performed at the CGC labs. The c.257C>T p.(Pro86Leu) was detected in heterozygosity in the *DPYD* gene (Table 1).

He subsequently underwent Lower anterior resection surgery. The pathology report indicated a complete response with no residual malignancies. Seventeen lymph nodes were examined and were all negative for malignancy. No adjuvant systemic treatment was offered to this patient, and he is currently under surveillance with no evidence of disease recurrence as per the last colonoscopy and CT scan.

## 5. Methods

*DPYD* gene sequencing was performed as follows: exons and eight base pairs of intron-exon boundaries of the *DPYD* gene (NM_000110.3; chr.1) were amplified by polymerase chain reaction. Performed by tagmentation (QXT, Agilent Technologies, Santa Clara, CA, USA) and next-generation sequencing (MiSeq, Illumina). The dragen workflow (Illumina) was utilized for variant call and alignment. The classification and reporting of the variants were performed according to the international recommendations [6].

## 6. Discussion

Flouropyrimidines (FLPN) have been used in the treatment of many different types of cancer. Of the patients treated, 10%–30% experience severe treatment-related toxicity, which is lethal in 0.5%–1% of the patients. The most studied biochemical cause of intolerance to FLPN is a deficiency of the metabolic enzyme, DPD [7,8,9].

Approximately, up to 60% of the severe toxicities induced by FLPN can be attributed to DPYD mutations [10,11].

The *DPYD* gene is polymorphic with over a hundred mutations reported so far [12]. A few of those variants are the most extensively studied and strongly linked to DPD deficiency polymorphisms such as c.1905+1G>A, rs3918290 (∗2A), c.2846A>T, rs67376798 (D949V), I560S c.1679T>G (∗13), rs55886062, c.1129–5923C>G, and rs75017182 (HapB3) [4]. These variants are most abundant in Caucasians and clinically validated through genotype-guided dosing adjustment as per Clinical Pharmacogenetics Implementation Consortium (CPIC) [4].

The DPYD variant identified in Case 1 is a novel mutation reported and published previously by Bukhari et al. Case 1 is the first report, to the best of our knowledge, to identify the c.2434G>A, rs371313778 polymorphism in a patient with severe toxic effects secondary to standard dose of a 5-FU-based regimen [13].

Case 2 carried the polymorphism c.1601G>A p.(Ser534Asn), also known as DPYD*4 is a rare mutation identified previously and linked to clinical DPD deficiency [14,15,16]. This case represents an intermediate metabolizer despite being homozygous for DPYD*4.

Case 3 was heterozygous for the very rare c.257C>T (p. Pro86Leu) variant which was also reported prior to associate with clinical DPD deficiency. His Oncologist elected not to proceed with capecitabine after discovering this variant [15,16,17,18].

Based on the treatment course of reported cases, we believe carriers of these polymorphisms may behave clinically like intermediate metabolizers. Therefore, a reduced starting dose followed by further titration based on toxicity, or even therapeutic drug monitoring may be a preferred approach in patients harboring those polymorphisms [4]. This practice was based on the Clinical Pharmacogentics Implementation Consortium (CPIC) recommendations [4].

There are genetic biomarkers that were previously investigated like the thymidalate synthetase (TYMS) gene polymorphisms [19]. Another promising phenotypic biomarker is pretreatment uracil, which is elevated in a proportion of patients with DPD deficiency. Meulendijks et al. concluded that a uracil level of more than 16 ng mL^−1^ is strongly linked to 5-FU severe toxicities. This study also found that pretreatment uracil of 16 ng mL^−1^ is strongly correlated with certain polymorphisms like DPYD*2, DPYD *13 and DPYD *9B [7].

Based on accumulating evidence, we believe pre-emptive pharmacogenomic testing will help with selecting the most appropriate treatment and dosing, thus reducing the potentially severe and lethal side effects. Cost-effectiveness studies are needed and will potentially expediate the implementation of pharmacogenomic-based practice in this field [3,19,20]. To our knowledge, this is the first case series from Saudi Arabia looking at DPYD polymorphisms in patients with severe toxicity to 5-FU- based chemotherapy.

## 7. Conclusions

This case series clearly demonstrates the benefit of DPYD gene sequencing in detecting rare variants in our population for whom there is limited pharmacogenetic knowledge. It also illustrates that genotype-based dosing in the presence of c.2434G>A, c.1601G>A and c.257C>T. DPYD variants are effective for preventing deleterious 5-FU side effects. We will need more studies to determine the cost-effectiveness of gene sequencing in this setting. Other genotypic and phenotypic biomarkers such as TYMS variants screen and pretreatment uracil level could serve as potential predictors of toxicity however need further clinical validation.

## Figures and Tables

**Table 1:** Patients clinical characteristics, polymorphisms identified and treatments received.

Case	Age	Gender	Chemotherapy Received	Manifestations	5-FU or Capectabine Dose-Modification	DPYD Polymorphism
Case 1	65	F	FOLFOX	G3 mucositis, diarrhea, and pancytopenia	40% dose-reduction	Heterozygous for c.2434G>A. Amino acid change: p.Val812lle
Case 2	64	F	XOLOX (Capecitabine starting dose 1000 mg/m^2^ twice daily)	G3 mucositis, diarrhea, and pancytopenia	40% dose-reduction	Homozygous for c.1601G>A. Amino acid change: p.(Ser534Asn)
Case 3	66	M	Capecitabine with radiation (RT). (Capecitabine dose at 625 mg/m^2^ twice daily on RT days)	G3 diarreha and GIV neutropenia	Capecitabine was stopped on week 4 of CCRT	Heterozygous for c.257C>T. Amino acid change: p.(Pro86Leu)
